# Single-cell genomics identifies distinct B1 cell developmental pathways and reveals aging-related changes in the B-cell receptor repertoire

**DOI:** 10.1186/s13578-022-00795-6

**Published:** 2022-05-07

**Authors:** Yao Luo, Jing Wang, Kairui Li, Mingxia Li, Shasha Xu, Xingjie Liu, Zhiwei Zhang, Xiang Xu, Yu Zhang, Jiawei Pan, Pengtao Liu, Shaorong Gao, Zhichao Miao, Yong Yu

**Affiliations:** 1 Shanghai Key Laboratory of Maternal Fetal Medicine, Clinical and Translational Research Center of Shanghai First Maternity and Infant Hospital, Frontier Science Center for Stem Cell Research, School of Life Sciences and Technology, Tongji University, 1239 Siping Road, Shanghai, 200092 China; 2 Translational Research Institute of Brain and Brain-Like Intelligence and Department of Anesthesiology, Shanghai Fourth People’s Hospital Affiliated to Tongji University School of Medicine, Shanghai, 200081 China; 3 European Bioinformatics Institute, European Molecular Biology Laboratory, Wellcome Genome Campus, Hinxton, Cambridge, CB10 1SD UK; 4 Guangzhou Laboratory, Guangzhou International Bio Island, Guangzhou, 510005 Guangdong China; 5 School of Biomedical Sciences, Li Ka Shing Faculty of Medicine, Stem Cell and Regenerative Medicine Consortium, The University of Hong Kong, 5 Sassoon Road, Pokfulam, Hong Kong; 6 Laboratory Animal Research Center, School of Medicine, Tongji University, Shanghai, 200092 China

**Keywords:** B1 cell development, scRNA-seq, scBCR-seq, B1 cell aging, B1 cell maintenance

## Abstract

**Background:**

B1 cells are self-renewing innate-like B lymphocytes that provide the first line of defense against pathogens. B1 cells primarily reside in the peritoneal cavity and are known to originate from various fetal tissues, yet their developmental pathways and the mechanisms underlying maintenance of B1 cells throughout adulthood remain unclear.

**Results:**

We performed high-throughput single-cell analysis of the transcriptomes and B-cell receptor repertoires of peritoneal B cells of neonates, young adults, and elderly mice. Gene expression analysis of 31,718 peritoneal B cells showed that the neonate peritoneal cavity contained many B1 progenitors, and neonate B cell specific clustering revealed two trajectories of peritoneal B1 cell development, including pre-BCR dependent and pre-BCR independent pathways. We also detected profound age-related changes in B1 cell transcriptomes: clear difference in senescence genetic program was evident in differentially aged B1 cells, and we found an example that a B1 subset only present in the oldest mice was marked by expression of the fatty-acid receptor CD36. We also performed antibody gene sequencing of 15,967 peritoneal B cells from the three age groups and discovered that B1 cell aging was associated with clonal expansion and two B1 cell clones expanded in the aged mice had the same CDR-H3 sequence (AGDYDGYWYFDV) as a pathogenically linked cell type from a recent study of an atherosclerosis mouse model.

**Conclusions:**

Beyond offering an unprecedent data resource to explore the cell-to-cell variation in B cells, our study has revealed that B1 precursor subsets are present in the neonate peritoneal cavity and dissected the developmental pathway of the precursor cells. Besides, this study has found the expression of CD36 on the B1 cells in the aged mice. And the single-cell B-cell receptor sequencing reveals B1 cell aging is associated with clonal expansion.

**Supplementary Information:**

The online version contains supplementary material available at 10.1186/s13578-022-00795-6.

## Introduction

B lymphocytes contribute to both humoral and cellular immunity, serving as antibody-producing and antigen-presenting cells [1]. B cells are generally divided into two major subsets: B1 and B2 cells. B2 cells generate the well-described germinal center and T-cell-dependent immune responses against protein antigens, whereas B1 cells produce a rapid T-cell-independent antibody response—primarily targeting lipids and polysaccharides [2, 3]. In mouse, B1 cells primarily reside in body cavities including the peritoneal cavity—a fluid-filled space located between the wall of the abdomen and organs [4, 5]. B1 cells secrete most of the circulating natural antibodies, including those which recognize endogenous (i.e., self) antigens detectable on apoptotic cells and oxidized lipids [6–8], natural anti-influenza virus IgM antibodies [9], and gut mucosa protective IgA antibodies [10]. B1 cells also secrete immune regulatory cytokines including interleukin-10 [11] and granulocyte-monocyte colony stimulating factor [12]. Both regulatory cytokines and natural antibodies produced by B1 cells are essential for maintaining physiological and immune homeostasis [3, 4]. Furthermore, peritoneal B1 cells have been shown to exert phagocytic and microbicidal abilities [13].

Recent studies have revealed that B1 cell precursors are distinct from B2 cell precursors in terms of their distinct transcriptional signature and divergence in their networks of transcription factors governing specification and fate commitment [14, 15]. B1 cell development occurs in two fetal waves and one adult wave [15, 16], whereas B2 cells are generated and continuously replenished via hematopoiesis in adults. Precursors of B1 cells have been detected in diverse tissues, including the yolk sac [17], para-aortic splanchnopleure [18], aorta-gonad-mesonephros [19], placenta [20], fetal and neonatal liver [21], fetal omentum [22–24], spleen [25], and bone marrow [26]. While B1 cell progenitors are present in adult bone marrow, several lines of evidence indicate that their contribution to adult B1 cell pool is quite limited under physiological condition [26–29]. A recent lineage tracing experiment with the hematopoietic stem cell restricted Pdzk1ip1-CreER line in tamoxifen-treated adult mice revealed that the contribution of adult hematopoiesis to B1 cell subset is below 10% over a 40-week observation period [29].

Given the various origins of B1 cell precursors and multiple development waves, it follows that the postnatal B1 cell pool must be a heterogeneous population, and likely one that differs substantially with aging. We do know that B1 cells are maintained by self-renewal in adults [27, 30–32] and their functions are known to be affected by aging [33–39]. Of particular note, a report revealed that passive transfer of IgG-depleted serum from elderly (18–24 months) mice had no effect on immune-deficient CB17-SCID mice which infected with streptococcus pneumoniae, whereas IgG-depleted serum from young adult (3 months) mice was protective [39]. This result clearly demonstrates that the protection against pneumococcal infection afforded by natural IgM antibodies in vivo diminishes with age. Another well-known age-related phenomenon with B1 cells is their general expansion in senescent mice [33]. However, much remains unclear about the mechanisms underlying many of these differences observed in aged mice.

We also know that the B-cell receptor (BCR) repertoire of B1 cells is affected by aging: dynamic changes in the B1 cell BCR repertoire during aging were recently profiled by sequencing transcripts of the complementarity determining region 3 of the heavy chain (CDR-H3) [40]. This elegant study revealed that the BCR repertoire of B1 cells is continuously shaped by stringent selection throughout the lifetime of a mouse, and becomes progressively restricted with age. However, this study was conducted using bulk sequencing approaches, which required pre-sorting of B cell subsets and which limited their capacity to characterize cell-to-cell variations in the BCR repertoire of aging B1 cells.

Here, using single-cell RNA-sequencing (scRNA-seq) of peritoneal CD19^+^ cells from neonates, young adults, and elderly mice, we revealed that a large majority of peritoneal B cell subsets were B1 cells in adults. We found that neonate peritoneal B1 cell progenitors develop along two distinct trajectories towards B1 cells, including a pre-BCR dependent pathway and a pre-BCR independent pathway. Aging-related changes were obvious in the transcriptomes of B1 cells from the variously aged mice, and we discovered that a subset only present in the elderly mice could be easily sorted based on the surface marker CD36. Subsequently, single-cell B-cell receptor sequencing (scBCR-seq) of peritoneal CD19^+^ cells from the variously aged mice experimentally tracked how the BCR repertoire of peritoneal B1 cells became progressively restrictive with age. Moreover, our scBCR-seq data also support profiling of B1 cell clonal expansion, and we detected the accumulation of cells with an atherosclerosis-associated CDR-H3 sequence.

## Results

### A large majority of B cells in the mouse peritoneal cavity are B1 cells

**Fig. 1:**
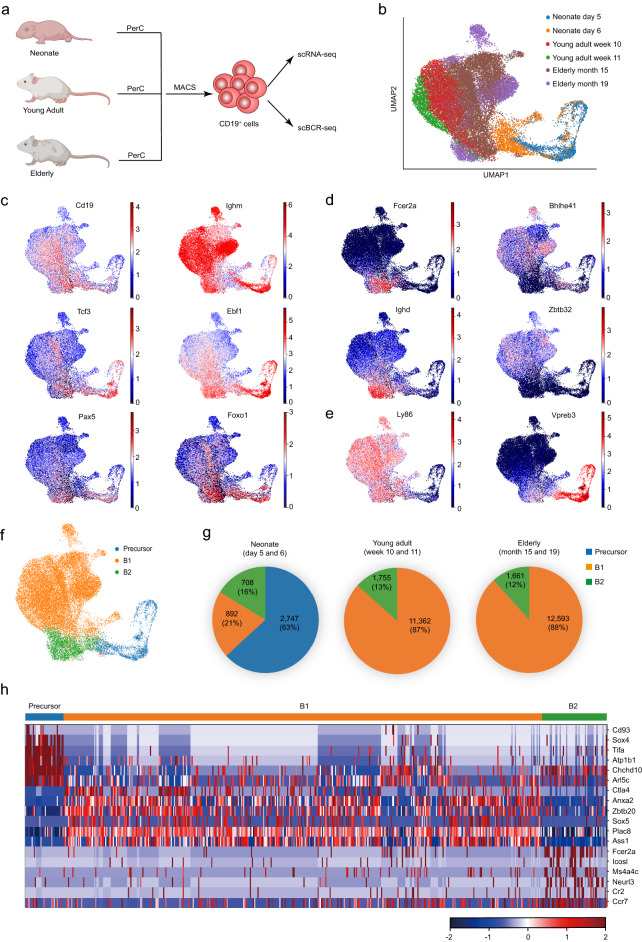
scRNA-seq analysis of peritoneal B cells in neonatal, young, and elderly mice. **a** Experimental design for scRNA-seq and scBCR-seq analysis of peritoneal B cells (created with BioRender). **b** A Uniform Manifold Approximation and Projection (UMAP) visualization of scRNA-seq data of all examined peritoneal CD19^+^ cells. Cells were purified from neonates (day 5, pool of 10 pups from 1 litter; day 6, pool of 7 pups from 1 litter), young adults (week 10, n = 1; week 11, n = 2), and elderly (month 15, n = 1 and month 19, n = 1) of wildtype C57BL/6 mice. **c**−**e** Distribution of gene expression in UMAP plots. The color key indicates the expression level. **f** Distribution of B1 cells, B2 cells, and B cell precursors in the UMAP plot. **g** The pie charts show the percentages of peritoneal B1 cells, B2 cells, and B cell precursors in different ages. **h** The heatmap shows the expression patterns of indicated genes in B1 cells, B2 cells, and B cell precursors. The color key indicates the expression level. PerC, peritoneal cavity. MACS, magnetic-activated cell sorting

In order to investigate the B1 cell development in the peritoneal cavity, we purified individual CD19^+^ cells in the peritoneal cavity by magnetic-activated cell sorting from neonates, young adults, and elderly mice for scRNA-seq and scBCR-seq analysis (Fig. 1a). After sequencing quality control (Additional file 1: Fig. S1a−d) and depletion of the contaminated non-B cells (Additional file 1: Fig. S1e−g), we analyzed 31,718 B cells derived from mice of the three age groups (Fig. 1b). Dimensional reduction analysis by uniform manifold approximation and projection (UMAP) revealed that almost all cells expressed the known B cell regulators including *Cd19*, *Ighm*, *Tcf3*, *Ebf1*, *Pax5,* and *Foxo1* (Fig. 1c), confirming that the examined cells were indeed B cells. *Fcer2a* (which encodes CD23) and *Ighd* were used as markers to distinguish B2 from B1 cells [4]. Both *Fcer2a* and *Ighd* were highly expressed in the left bottom subset of the UMAP projection (Fig. 1d), while the B1 cell genes *Bhlhe41* [41, 42] and *Zbtb32* [42, 43] were highly expressed in most of the other cells (Fig. 1d).

The mutually exclusive expression of the mature B cell gene *Ly86* [44] and B cell precursor gene *Vpreb3* [45] suggested the presence of both mature B cells and their precursors in examined CD19^+^ cells (Fig. 1e). As mentioned above, the mature peritoneal B cells contain B1 cells (*Bhlhe41*^+^*Fcer2a*^*−*^) and B2 cells (*Bhlhe41*^*−*^*Fcer2a*^+^). Thus, the peritoneal CD19^+^ cells were roughly grouped into 3 clusters: B1 cells, B2 cells, and B cell precursors (Fig. 1f; Additional file 2: Table S1). Interestingly, all B cell precursors were sourced from neonates and accounted for 63% of the total peritoneal B cells of neonatal mice (Fig. 1g). The mature B1 cells accounted for 21% in neonatal mice, while their ratio increased to 87% in young adults and 88% in elderly mice (Fig. 1g).

In addition to the reported B cell regulators *Sox4* [46] and *Cd93* [47], we discovered potential new genes that were expressed in peritoneal B cell precursors, for example, *Tifa*, *Atp1b1*, *Chchd10*, and *Arl5c* (Fig. 1h). Our scRNA-seq analysis also revealed additional B1 cell genes including *Ctla4*, *Anxa2*, *Zbtb20*, *Sox5*, *Plac8*, and *Ass1*, as well as B2 cell genes such as *Icosl*, *Ms4a4c, Neurl3*, *Cr2*, and *Ccr7* (Fig. 1h). These scRNA-seq results for neonates, young adults, and elderly mice show that a large majority of B cells in the mouse peritoneal cavity are B1 cells and that B cell precursors are present in the neonate peritoneal cavity.

### The neonate peritoneal cavity contains a high proportion of B1 cell progenitors

**Fig. 2:**
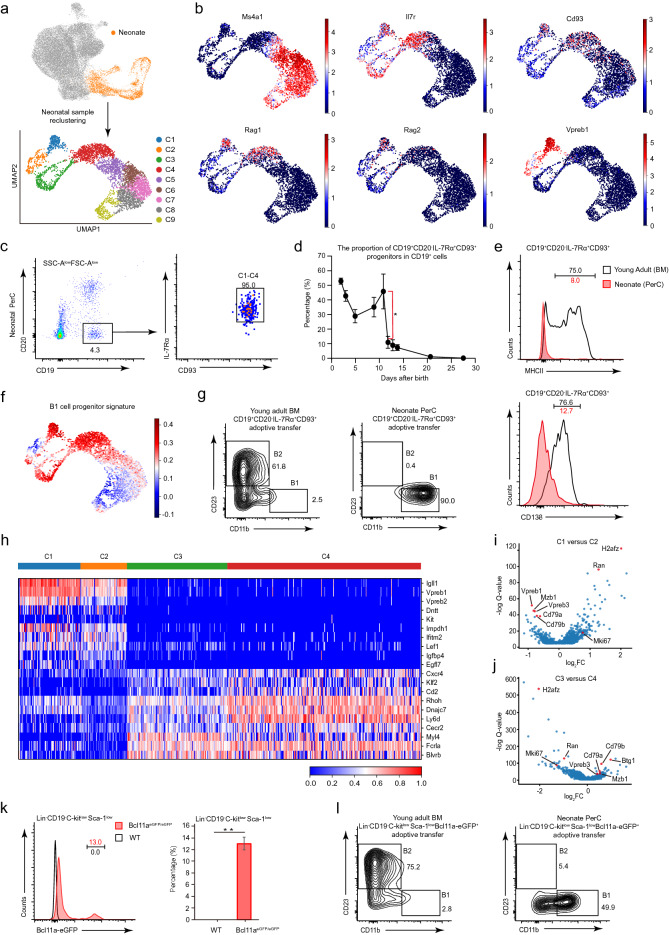
Characterization of B1 progenitors in the neonate peritoneal cavity. **a** The neonatal cells were highlighted in the initial UMAP projection, and they were re-projected into nine clusters (C1-C9) based on Harmony corrected latent space. **b** Distribution of gene expression in UMAP plots. The color key indicates the expression level. **c** Neonate peritoneal CD19^+^CD20^−^IL-7Rα^+^CD93^+^ B cell progenitors were analyzed by flow cytometry. Neonates (day 5, pool of 7 pups from 1 litter) were used.** d** The dynamic change of the proportion of CD19^+^CD20^−^IL-7Rα^+^ CD93^+^ B cell progenitors during the first few weeks after birth (n = 3 per time point). The error bars indicate the s.e.m. *P < 0.05. **e** FACS plots showing expression of MHCII molecules and CD138 in CD19^+^CD20^−^IL-7Rα^+^CD93^+^ B cell progenitors. Neonates (day 5–6, n = 3) and young adult mice (week 8–12, n = 3) were used in the analysis. **f** Projection of the B1 cell progenitor signature (B1 cell progenitor signature was from a previous study [15], see more details in Methods). The color key indicates the average log normalized expression of the B1 signature genes over the randomly sampled reference genes. **g** The FACS plots showing the adoptive transfer results. 2–4 weeks after injection of indicated cells directly into the peritoneal cavity of *Rag2*^*−/−*^ recipient mice (n = 3 for each donor cell type), peritoneal cells of recipient mice were isolated and analyzed by flow cytometry. Neonates (day 5–6, pool of 25 pups from 3 litters) and young adult mice (week 8–12, n = 2) were used as donors. **h** Heat map of the expression levels of the indicated genes in C1, C2, C3, and C4 cells. The color key indicates the expression level. **i** Volcano plot showing the differentially expressed genes (DEGs) between C1 and C2 cells. DEGs (FDR < 0.01) were plotted. **j** Volcano plot showing the differentially expressed genes (DEGs) between C3 and C4 cells. DEGs (FDR < 0.01) were plotted. **k** FACS plots showing the expression of Bcl11a in neonate peritoneal Lin^−^CD19^−^C-kit^low^Sca-1^low^ cells. Neonates (day 6, pool of 12 pups from 2 litters, n = 3) were used. The error bars indicate the s.e.m. **p < 0.01. **l** FACS plots showing the adoptive transfer results. 2–4 weeks after intravenous injection of indicated cells into *Rag2*^*−/−*^ recipient mice (n = 3 for each donor cell type), peritoneal cells of recipient mice were isolated and analyzed by flow cytometry. Neonates (day 6, pool of 26 pups from 3 litters) and young adult mice (week 8–12, n = 2) were used as donors. Data are representative of three (**c**, **d**, **g**, **k**, **l**) independent experiments

To analyze peritoneal B cell precursors in early life, we re-clustered the neonatal samples (Fig. 2a). A distinct aggregation pattern of B cells was observed in the re-clustered UMAP projection, wherein all of the neonatal cells were grouped into nine clusters (Fig. 2a and Additional file 1: Fig. S2a and Additional file 2: Table S1)*.* The cells in clusters 1, 2, 3, and 4 (denoted as C1-C4) expressed no detectable *Ms4a1* (which encodes CD20) but expressed *Il7r* and *Cd93* (Fig. 2b). C1 and C4 cells expressed the known DNA recombination genes *Rag1* and *Rag2* (Fig. 2b). C1-C2 cells expressed a pre-BCR surrogate light chain gene *Vpreb1* (Fig. 2b).

The expression of the early B cell genes suggested that C1-C4 cells were early B cell progenitors. To experimentally validate neonate peritoneal B cell progenitors, we performed flow cytometry analyses. Consistent with scRNA-seq data, C1-C4 cells were identified as CD19^+^CD20^−^IL-7Rα^+^CD93^+^ cells (Fig. 2c). These B cell progenitors occurred at a high frequency in the first few days after birth (until day 11), after which their frequency dramatically decreased (Fig. 2d). We also noted that B cell progenitors production ceased at about weaning age (Fig. 2d).

The lack of MHCII molecules and CD138 expression on fetal B1 progenitors distinguished them from bone marrow B2 progenitors [48–50]. C1-C4 cells expressed almost no detectable *H2-Aa* (encoding the MHCII molecules subunit), *Ciita* (MHCII molecules regulator), or *Sdc1* (which encodes CD138) (Additional file 1: Fig. S2b), and very few C1-C4 cells expressed MHCII molecules or CD138 on their surface (Fig. 2e), suggesting a B1 cell progenitor identity. Moreover, scoring the single cells with the signatures of C1-C9 clusters revealed that C1, C3, C4 and most of C2 cells expressed B1 cell progenitor signature (Fig. 2f and Additional file 1: Fig. S2c). Pursuing this, we sorted C1-C4 progenitors from neonates and adoptively transferred them into sub-lethally irradiated *Rag2*^*−/−*^ mice. Engrafted control adult bone marrow CD19^+^CD20^−^IL-7Rα^+^CD93^+^ progenitors mainly produced B2 cells, whereas neonate peritoneal CD19^+^CD20^−^IL-7Rα^+^CD93^+^ C1-C4 progenitors generated B1 cells (Fig. 2g).

C1-C2 cells expressed typical pro-B cell genes (*Igll1*, *Vpreb1*, *Vpreb2, Dntt,* and *Kit*), whereas C3-C4 cells had a pre-B cell signature (*Cxcr4*, *Klf2*, and *Rhoh*) (Fig. 2h). Comparing C1 with C2 cells, we observed C1 cells had higher expression levels of surrogate light chain genes (*Vpreb1*, *Vpreb3*) and Ig related genes (*Cd79a*, *Cd79b*, *Mzb1*) (Fig. 2i; Additional file 3: Table S2). C2 cells had a prominent proliferative signature marked by the expression of cell cycle regulators (*Mki67*, *H2afz*, *Ran*) (Fig. 2i; Additional file 4: Table S3). Comparing cells of the C3 with C4 clusters, C3 cells were clearly marked by the expression of cell proliferation regulators (*Mki67*, *H2afz*, *Ran*), whereas C4 cells had relatively higher expression of the anti-proliferation gene *Btg1* and the Ig-associated gene (*Cd79a*, *Cd79b*, *Mzb1*, *and Vpreb3*) (Fig. 2j; Additional file 5: Table S4; Additional file 6: Table S5). Interestingly, we discovered many potential cluster-specific genes (Additional file 1: Fig. S2d, e): for example, *Fcrl6*, *Speer2*, and *Selenom* (in C1 cells); *Bok*, *Dnph1*, *Impa2* (in C2 cells); *Rgs16*, *Serinc5*, and *Il2ra* (in C4 cells).

Successive developmental stages of bone marrow B2 cells can be distinguished based on the correlated expression of various cell surface markers [51]. We investigated similar surface markers to experimentally identify C1, C2, C3, and C4 cells. Within the neonate peritoneal CD19^+^CD20^−^IL-7Rα^+^CD93^+^ cell population, the C1-C2 cells were C-kit^+^CD43^+^, and could be further divided into Ki67^−^ C1 cells and Ki67^+^ C2 cells. C3 cells were Ly6d^+^CD25^−^Ki67^+^, and C4 cells were Ly6d^+^CD25^+^Ki67^−^ (Additional file 1: Fig. S2f, g and 3d).

A previous *Bcl11a*^*eGFP*^ reporter mice study showed that the transcription factor Bcl11a is highly expressed in early lymphoid progenitors [52]. Using this mouse reporter line, we found a subset of Lin^−^CD19^−^C-kit^low^Sca-1^low^ cells which expressed a high level of Bcl11a in the neonate peritoneal cavity (Fig. 2k and Additional file 1: Fig. S2h). We isolated these cells from the *Bcl11a*^*eGFP*^ reporter mice for adoptive cell transfer assays. In contrast to engrafted control adult bone marrow Lin^−^CD19^−^C-kit^low^Sca-1^low^ Bcl11a-eGFP^+^ cells mainly generating B2 cells, engrafted neonate peritoneal Lin^−^CD19^−^C-kit^low^Sca-1^low^Bcl11a-eGFP^+^ cells produced B1 cells (Fig. 2l), demonstrating that early lymphoid progenitors with B1 cell potential are present in the peritoneal cavity of neonate mice.

### Single-cell analysis reveals two trajectories of B1 cell development in neonate peritoneal cavity

**Fig. 3:**
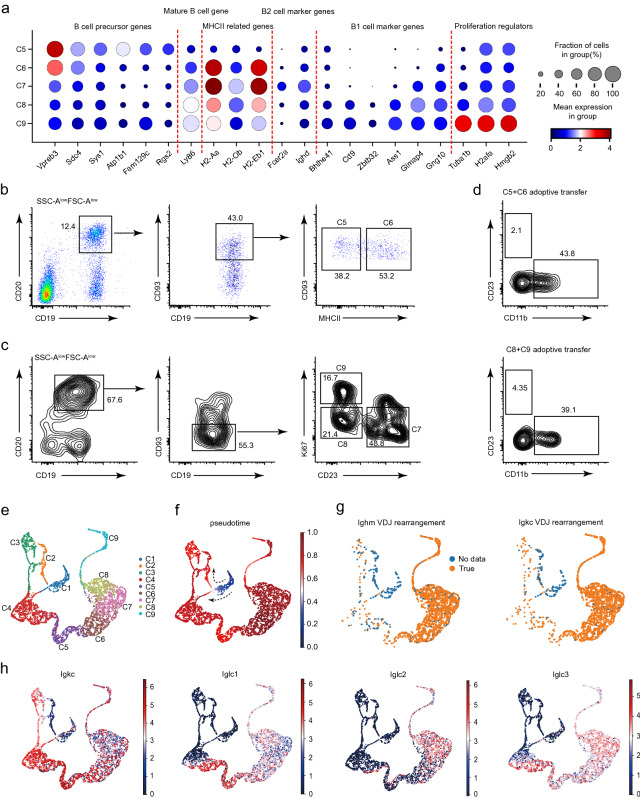
Dissecting the trajectory of neonate peritoneal B1 cell development. **a** The dot plot demonstrates marker genes expression in the C5-C9 clusters. **b**, **c** FACS plots validate that C5-C9 clusters are present in the neonate peritoneal cavity. Neonates (day 5–6, pool of 16 pups from 2 litters) were used for analysis. **d** FACS plots show the adoptive transfer results. Two weeks after injection of indicated cells directly into the peritoneal cavity of *Rag2*^*−/−*^ recipient mice (n = 3 for each donor cell type), peritoneal cells of recipient mice were isolated and analyzed by flow cytometry. Neonates (day 5–6, pool of 14 pups from 2 litters) were used as the donors. **e** The Palantir-derived UMAP plot shows another visualization of C1-C9 cells. **f** The diffusion pseudotime of C1-C9 cells was analyzed by Palantir (see Methods). The color key indicates the pseudotime from early (blue) to late (dark red). **g** UMAP plots showing the V(D)J rearrangement status of *Igh* and *Igk* chains in C1-C9 cells (see Methods). True means that the expression of rearranged V(D)J gene segments was captured by the B-cell receptor sequecning. Neonates (day 6, pool of 7 pups from 1 litter) were used for B cell receptor sequecning analysis. **h** Distribution of κ and λ light chain constant region gene expression in the Palantir-derived UMAP plot. The color key indicates the expression level. Data are representative of two (**b** and **c**) or there **d** independent experiments

C5 and C6 cells expressed the known B cell precursor genes *Vpreb3* and *Cd93* (Fig. 2b, 3a)*,* whereas C7-C9 cells expressed the mature B cell gene *Ly8*6 and multiple MHCII molecules related genes (*H2-Aa*, *H2-Ob*, *H2-Eb1*) (Fig. 3a and Additional file 1: Fig. S3b). Interestingly, these MHCII molecules related genes were also highly expressed in C6 cells, but were very low in C5 cells (Fig. 3a and Additional file 1: Fig. S3b), enabling discernment of C6 cells from C5 cells. Finally, we noted that C7 cells expressed relatively high levels of B2 cell marker genes (*Fcer2a* and *Ighd*), while C8-C9 cells expressed B1 cell marker genes (*Bhlhe41* and *Cd9*) (Fig. 3a and Additional file 1: Fig. S3c); and C8 and C9 cells were well demarcated by their expression of proliferation regulators (*Tuba1b*, *H2afa*, *Hmgb2*) (Fig. 3a).

We next performed flow cytometry and adoptive cell transfer analyses with C5-C9 cells. Consistent with our scRNA-seq data, C5 cells were identified as CD19^+^CD20^+^CD93^+^MHCII^−^ cells and C6 cells were CD19^+^CD20^+^CD93^+^MHCII^+^ (Fig. 3b). C7 cells were CD19^+^CD20^+^CD93^−^CD23^+^, C8 cells were CD19^+^CD20^+^CD93^−^CD23^−^Ki67^−^, and C9 cells were CD19^+^CD20^+^CD93^−^CD23^−^Ki67^+^ (Fig. 3c and Additional file 1: Fig. S3d). Notably, transplantation assays demonstrated that C5, C6, C8, and C9 cells produced B1 cells, but not B2 cells in vivo (Fig. 3d).

We performed pseudotime analysis of our neonate B cell data using Palantir to help understand the relationship(s) between developmental stages and to ascertain a peritoneal B1 cell development trajectory [53]. According to the obtained pseudotime values, C1 cells appear to represent the beginning stage for B1 cell development among CD19^+^ cells (Fig. 3e, f). Analysis of the V(D)J recombination status of both Ig heavy and light chains showed that few cells in C1-C2 had completed recombination (Fig. 3g), confirming that C1-C2 cells represented the earliest B1 cell progenitors among the detected clusters. It was notable that only a few of the cells from the C1-C2 clusters expressed Ig light chain constant-region transcripts (*Igkc*, *Iglc1*, *Iglc2*, *Iglc3*) (Fig. 3h), findings suggesting that the majority of C1-C2 cells cannot initiate light chain rearrangement [54].

C3-C4 cells initially expressed *Igkc,* and some C4 cells expressed *Iglc1*, *Iglc2*, and *Iglc3* (Fig. 3h). C5-C9 cells appeared to accomplish for V(D)J combination of both Ig heavy and light chains, and express light chain constant-region transcripts (Fig. 3g, h). Interestingly, the pseudotime analysis suggested that C4 cells may differentiate directly from C1 cells (Fig. 3f). We noted that 5.6% (4/72) of C1 cells completed V(D)J combination of both Ig heavy and kappa chains and also expressed *Igkc* (Fig. 3g, h), suggesting that Ig heavy and light chain recombination may occur simultaneously in these cells. If so, this could result in functional BCR formation, which may bypass the pre-BCR dependent expansion and selection stages [55]. Taken together, our Palantir analysis supports the speculation that neonate peritoneal B1 cell development apparently comprises two trajectories: a pre-BCR dependent developmental trajectory (C1-C2 to C3-C4) as well as a pre-BCR independent developmental trajectory (C1 to C4).

### B1 cells exhibit profound age-related changes in their transcriptomes

**Fig. 4:**
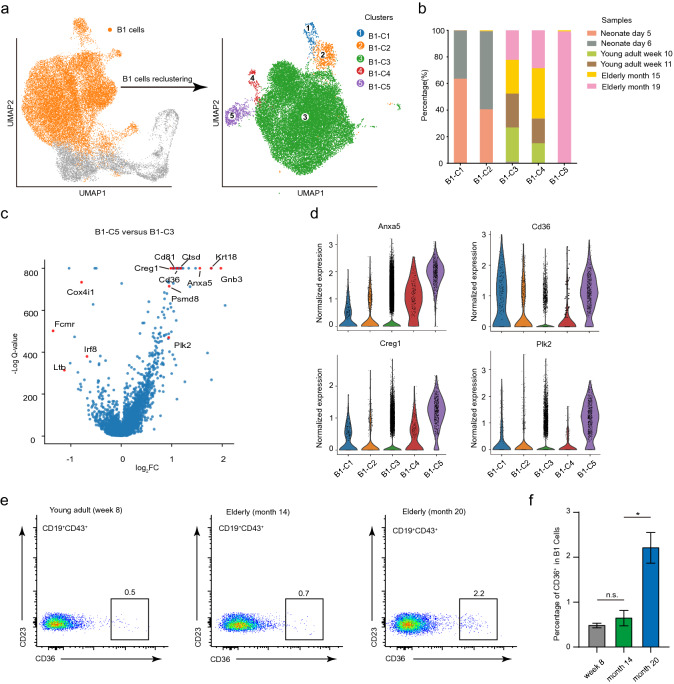
Characterizing the gene signature of aged B1 cells. **a** The mature B1 cells (*Bhlhe41*^+^*Fcer2a*^−^*Ly86*^+^) of the initial UMAP projection (highlighted in orange), which was re-grouped into five clusters (B1-C1 to B1-C5) based on Harmony corrected latent space. **b** Bar plot showing the distributions of cells from the five B1 clusters for the six indicated groups of differentially aged mice. **c** Volcano plot showing the differentially expressed genes (DEGs) between B1-C5 and B1-C3 cells, DEGs (FDR < 0.01) were plotted. The aging-related genes were highlighted in red. **d** Violin plots comparing expression of cellular senescence related genes in different B1 clusters. The y axis indicates the log normalized expression level. **e** FACS plots showing expression of CD36 in CD19^+^CD43^+^CD23^−^ B1 cells among the mice of indicated ages. Young adult (week 8, n = 3), elderly (month 14, n = 3) and elderly (month 20, n = 3). **f** The bar plot shows the statistics of the percentage of CD36^+^ cells in B1 cells in FACS analysis. The error bars indicate the s.e.m. *P < 0.5. n.s. not significant

In addition to our focused study of the neonate-specific subgroup of peritoneal CD19^+^ cells, we also re-clustered mature B1 cells (*Bhlhe41*^+^*Fcer2a*^*−*^*Ly86*^+^) presenting in mice of all the sampled age groups (Fig. 4a). This generated five clusters of B1 cells which we denoted as B1 cell clusters 1–5 (B1-C1 to B1-C5) (Fig. 4a; Additional file 2: Table S1). Perhaps the most obvious trend was that these clusters were differentially enriched in mice of the different age groups (Fig. 4b). That is, the large majority of the total cells from B1-C1 and B1-C2 were from the neonate mice (both the day 5 and 6 groups) (Fig. 4b). In contrast, the vast majority of the B1-C5 cells were found in the most advanced age group of mice (Fig. 4b and Additional file 1: Fig. S4a). The B1-C3 and B1-C4 cells were variously present in young adults and in the elderly mice (Fig. 4b).

Many previous studies have shown that young B1 cells exert different functions than aged B1 cells in a variety of disease contexts, including *streptococcus pneumonia* infection [39, 56], atherosclerosis [57, 58], and insulin resistance [38]. We therefore used our B1 cells clusters to explore gene expression signatures characteristic of differentially aged B1 cells. Comparing B1-C5 cells against the other non-proliferating adult B1 cells, we detected 1,468 upregulated DEGs in C5 cells, including 71 cellular senescence-related genes by a Reactome [59] pathway analysis (Fig. 4c and Additional file 1: Fig. S4b; Additional file 7: Table S6; Additional file 8: Table S7). Notably, the known cellular senescence marker genes such as *Anx5a* [60], *Cd36* [61], *Creg1* [62] and *Plk2* [63] were expressed higher in B1-C5 cells (Fig. 4c, d and Additional file 1: Fig. S4c). Interestingly, the surface molecule CD36 is highly enriched in B1-C5 cluster (Fig. 4d), we confirmed this while also demonstrating that CD36 can be used to easily isolate cells of this aged B1 cluster via flow cytometry (Fig. 4e, f). We anticipate that this surface marker will greatly facilitate studies of the aging-related B cell dysfunction generally and in several specific disease contexts.

### B1 cell aging is associated with clonal expansion

To characterize aging-related changes in the B-cell receptor repertoire of peritoneal B1 cells we performed scBCR-seq; that is, high-throughput single-cell B-cell receptor sequencing to obtain the paired full-length variable regions of heavy (V_H_) and light (V_L_) chains that determine the antigen specificity of antibodies [64]. The single-cell resolution data allowed us to characterize BCR diversity on B1 cells, to identify particular B1 cell clones, and to quantify their expansion based on the numbers of cells for each clone.

**Fig. 5:**
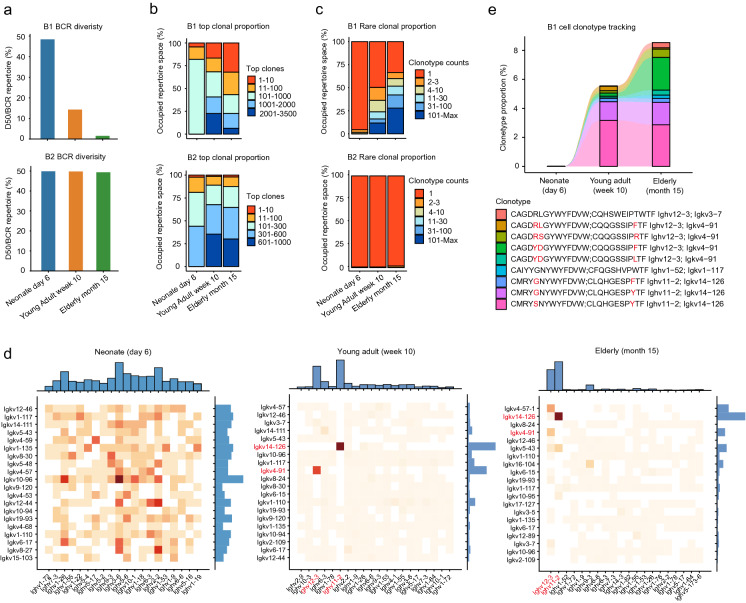
scBCR-seq reveals the clonal expansion of B1 cell with age. **a** The bar plot of the D50 metric quantifying the paired V_H_-V_L_ amino acid sequences (see Methods). **b** The stacked bar plot shows the distribution of the top clonal proportions of both B1 and B2 cells of differentially aged mice. The colors represent different clonotype indices, clonotypes were ranked by the clonotype counts. **c** The stacked bar plot shows the distribution changes of the rare clonal proportions of both B1 and B2 cells of differentially aged mice. The colors represent different clonotype counts. **d** Variable germline gene segment pairing for B-cell receptor repertoires of B1 cells from differentially aged mice. Heatmaps show the percentage of B1 cell clones with a particular V_H_-V_L_ pairing. Row and column histograms indicate marginal V_H_ and V_L_ frequencies, respectively. The top 20 enriched V_H_ and V_L_ genes were shown in each age. **e** The clonotype tracking bar plot shows the dynamic change of the specific B1 cell clones’ proportions with age. The BCR repertoire was analyzed by the R package immunarch [100] (see Methods)

We first characterized the BCR diversity of peritoneal B1 cells with age. D50 metric analysis quantifying the paired V_H_-V_L_ amino acid sequence diversity revealed that the BCR repertoire of peritoneal B1 cells became progressively more restricted with advancing age (Fig. 5a). This phenomenon was in obvious contrast to peritoneal B2 cells, which stably maintained a diverse BCR repertoire at each of the examined ages (Fig. 5a). Consistent with our D50 analysis, the repertoire space occupied by the top 10 clones of B1 cells progressively increased with age, whereas that repertoire space of peritoneal B2 cells was stably maintained (Fig. 5b). Clonotype counting showed that almost all of the peritoneal B2 cell clones comprised individual cells, and this remained true as mice age (Fig. 5c). In contrast, the percentage of peritoneal B1 cell clones representing individual cells gradually decreased with age (Fig. 5c), and this decrease was accompanied by a concomitant increase in the proportion of B1 cell clones comprising many cells (> 100 cells) (Fig. 5c).

Given the BCR repertoire of peritoneal B1 cells becomes more restricted with age, we asked whether the identified B1 cell clones showed preferential usage of particular V_H_ genes, V_L_ genes, and/or V_H_-V_L_ gene pairings. We observed broad usage of both V_H_ and V_L_ genes by young adult and elderly B1 cells (Additional file 1: Fig. S5a). However, despite the broad V_H_ and V_L_ gene usages, certain V_H_-V_L_ gene pairings—notably *Ighv12-3/Igkv4-91* and *Ighv11-2/Igkv14-126*—were obviously enriched in young adult (~ 20%) and elderly (~ 24%) B1 cells (Fig. 5d and Additional file 1: Fig. S5b). In contrast, no obviously biased usage of V_H_ genes_,_ V_L_ genes_,_ or V_H_-V_L_ gene pairings was detected in neonatal B1 cells (Fig. 5d and Additional file 1: Fig. S5b).

Collectively, these data support that strong enrichment for certain adult B1 cell clones reflects clonal expansion with age [40, 65]. Indeed, clonotype tracking analysis revealed a number of B1 cell clones which obviously expanded with age (Fig. 5e). Of particular note, we found that seven of nine expanded B1 cell clones expressed either *Ighv12-3/Igkv4-91* or *Ighv11-2/Igkv14-126* pairing (Fig. 5e), both of which are known to recognize the phosphatidylcholine (PtC) present in plasma membranes of self-cells [66]. Moreover, B1 cells that marked by the expression of cell proliferation regulators (*Mki67*, *H2afz*, *Ran*) were highly enriched with anti-PtC B1 cell clones in both young adult (~ 43%) and elderly (~ 36%) mice (Additional file 1: Fig. S5c, d). The proliferation capacities of anti-PtC B1 cell clones in young adult and elderly mice also reflected their clonal expansion phenomenon.

Therefore, our scBCR-seq results for neonates, young adults, and elderly mice provide direct evidence that the BCR repertoire of peritoneal B1 cells becomes progressively more restricted with age and demonstrates that certain self-reactive B1 cell clones accumulate with age.

## Discussion

Mouse peritoneal cavity contains many B1 cells [3, 4, 67, 68]. The fetal peritoneal cavities of both mice and humans were discovered as an enriched source of B1 cells three decades ago [22–24]. Single-cell study of peritoneal CD19^+^ B cells from neonates, young adults, and elderly mice here demonstrates that B1 cell represent a large majority (> 80%) of the total peritoneal B cells in unmanipulated wild-type C57BL/6 adult mice under physiological condition. The single-cell data also reveal B1 cell precursor subsets in the neonate peritoneal cavity and delineate distinct B1 progenitor developmental stages and pathways.

Neonate peritoneal cavity harbors several differentially developmental stages of B1 cell precursors, including a non-proliferating pro-B cell subset (C1), a proliferating pro-B cell subset (C2), a proliferating pre-B cell subset (C3), and a non-proliferating pre-B cell subset (C4) (Additional file 1: Fig. S3d). We have identified many potential cluster-specific genes, for example, Fc receptor-like 6 (*Fcrl6*) that was specifically expressed in non-proliferating pro-B cell cluster. Fcrl6 was recently demonstrated to be a marker for defining biologically distinct B cell progenitors [69]. Consistent with the observation that Fcrl6^+^ pro-B cells exhibited protracted differentiation and proliferation [69], our single-cell transcriptome analysis showed no detectable cell cycle regulators was expressed in those *Fcrl6*^+^ pro-B cells (C1). Instead, *Fcrl6*^+^ pro-B cells had a high expression of the recombination activating gene, *Rag1*. These findings support that *Fcrl6*^+^ pro-B cells were undergoing Rag-mediated V(D)J gene segments rearrangement that has been demonstrated to couple with cell cycle state [70]. Moreover, our pseudotime analysis indicates *Fcrl6*^+^ pro-B cells are in the earlier development stage comparing to proliferative *Fcrl6*^−^ pro-B cells (C2). Early B1 progenitor subsets characterization will facilitate the studies in understanding the transcriptional networks that control the identity and function of B1 cells.

In the classical pathway of bone marrow B2 cell development, the developing B cell precursors first rearrange the Ig heavy-chain locus in pro-B cells, which results in the formation of pre-BCR and the expansion of large pre-B cells, and subsequently differentiate to small pre-B cells that rearrange the Ig light-chain (*Igk* or *Igl*) loci [51, 71, 72]. A recent study found that the fetal liver environment could promote a ‘premature’ *Igk* rearrangement at the pro-B cell stage leading to direct expression of mature BCRs at the surface, the B cell development in the fetal liver could bypass a requirement for pre-BCR dependent expansion and selection stage [55]. Taken together, these studies indicate that the bone marrow environment favors pre-BCR dependent B cell development, whereas the fetal liver supports pre-BCR independent B cell differentiation. Our pseudotime analysis suggests neonate peritoneal B1 precursors differentiation may follow two trajectories: a pre-BCR dependent developmental trajectory (C1-C2 to C3-C4) and a pre-BCR independent developmental trajectory (C1 to C4). Given that the neonatal period describes a time window that encompasses the ontogenetic transition from pre-BCR independent to pre-BCR dependent B cell development, it is reasonable to speculate that peritoneal B1 cells are likely produced by both pathways in neonates. If this speculation is correct, it will be interesting to determine which types of B1 cells are generated via a pre-BCR independent pathway versus those generated via a pre-BCR dependent pathway.

In addition, we discovered a Lin^−^CD19^−^C-kit^low^Sca-1^low^Bcl11a-eGFP^+^ lymphoid progenitors in neonate peritoneal cavity, which had the potential to differentiate into B1 cells, but not B2 cells upon transplantation. A recent fate-mapping study revealed that a transient developmentally restricted hematopoietic stem cell (drHSC) is the cell origin of innate-like B and T lymphocytes [73]. The drHSCs and Lin^−^CD19^−^C-kit^low^Sca-1^low^Bcl11a-eGFP^+^ lymphoid progenitors are both biased toward generating B1 cells and both of them are transiently present in the early life, indicating that Bcl11a marked peritoneal lymphoid progenitors might be differentiated from the drHSCs. Collectively, these findings support the insight that the B1 cell biased lymphoid progenitors might be already intrinsically committed to becoming B1 or B2 cells by the developmental stage before Ig chain rearrangement is initiated [74].

Our scRNA-seq analysis detected a small number of aged B1 cells in elderly mice. The identification of the surface molecule CD36 as a specific marker for aged B1 cells should be extremely helpful, enabling them to be isolated easily with flow cytometry for basic and potentially clinical investigations. While CD36^+^ B1 cells account for only a small fraction of peritoneal total B1 cells in advanced age mice, those cells can induce the senescence of nearby cells in a non-cell autonomous manner as demonstrated in a recent CD36 overexpression study [61]. CD36 is a scavenger receptor [75] and has been linked to the pathogenesis of atherosclerosis and Alzheimer’s disease through its recognition of modified endogenous ligands, including oxidized low-density lipoprotein (oxLDL) [76], fibrillar amyloid-β [77], and soluble amyloid-β [78]. Cd36-deficient mice are protected from the toxic effects of atherosclerosis [79], insulin resistance [80, 81], and fibrillar amyloid-β [82], so investigating the functions of CD36 of aged B1 cells, specifically in the context of those diseases, will likely yield etiopathologically and/or therapeutically relevant biological insights.

Our scBCR-seq analysis characterized the clonal expansion of B1 cells with advancing age in a highly resolved manner. Our data for neonatal mice shows that the BCR repertoire of peritoneal B1 cells is quite diverse, with a very large proportion of cells representing individual clones. In contrast, the BCR repertoire of peritoneal B1 cells in adult mice is restricted; the BCR repertoire of these cells is dominated by a few large B1 cell clones expressing particular BCRs (*e.g.*, *Ighv12-3/Igkv4-91* and *Ighv11-2/Igkv14-126*). Our data for both young adults and elderly mice enable us to detect an interesting trend: the extent of this BCR repertoire restriction increases with aging. The clonal outgrowth we observed is reminiscent of the behavior of cancer cells [83, 84], considering reports that transgenic mice expressing a B1 specific BCR spontaneously developed CLL in old age [85, 86], perhaps aged B1 cells may be prone to malignant transformation.

The clonal expansion of particular B1 cells may be relevant to other diseases: aged apoE-deficient mice with established atherosclerosis are known to harbor expansion of B1 cells with the CDR-H3 sequence AGDYDGYWYFDV [87]. Interestingly, the two B1 cell clones that were most expanded in our aged mouse samples have the same CDR-H3 sequence (Fig. 5e). Another study recently showed that the PtC binding BCR with the CDR-H3 sequence MRYGNYWYFDV can also bind oxLDL [88], and again B1 clones with this CDR-H3 were expanded in our aged mouse sample. The clonal expansion of similar B1 cell clones in aged mice and mice with established atherosclerosis motivates speculation about potential pathogenic links between atherosclerosis and B1 clonal expansion phenomena during aging.

## Conclusions

In summary, our combined scRNA-seq and scBCR-seq analyses of peritoneal B cells in differently aged mice enabled the identification of distinct B1 progenitor subsets, delineation of peritoneal B1 cell developmental trajectories, and characterization of distinct aged B1 subsets, as well as profiling of the age-related clonal evolution of the BCR repertoire in peritoneal B1 cells. Our study provides a high-resolution experimental basis and resource for studying the developmental heterogeneity and origins of B1 cells, and uncovers aging-related trends in the B cell distribution and BCR repertoire.

## Methods

### Mice

C57BL/6 wild-type mice were used for scRNA-seq and scBCR-seq. The *Bcl11a*^*eGFP*^ reporter mice were described in a previous study [52]. The *Rag2*^*−/−*^ mice (008,449) were purchased from the Jackson Laboratory. Neonates (day 5 and/or day 6), young adults (week 10 and/or week 11), elderly mice (month 15 and/or month 19) were used for scRNA-seq and scBCR-seq experiments. All mice used were maintained under a specific pathogen-free condition at the Laboratory Animal Research Center of Tongji University.

### Peritoneal B cells isolation and preparation for single-cell sequencing

Mice were sacrificed by cervical dislocation. Peritoneal B cells were isolated as previously described protocol [89]. Briefly, a small fold of skin was grasped at the umbilicus using forceps, and a small incision was made with a scissors. Then the opposing skin edges were pulled in opposite directions by fingers to expose the peritoneum of peritoneal cavity. Ice-cold DPBS (AF29584116, Hyclone) with 2% (volume/volume) FBS (S711001S, Lonsera) was injected into the abdominal cavity. The peritoneum was massaged gently and a needle with a syringe was inserted into the peritoneum to collect the fluid. Next, an incision was made in the inner skin of the peritoneum. The remaining fluid was aspirated gently with a pipette. The fluid was collected in the tube on ice, and was centrifuged for collecting peritoneal cells. The peritoneal B cells were enriched with CD19 Microbeads (130–090-301, Miltenyi Biotech). The selected individual CD19^+^ cells (~ 80–90% purity) from differently aged mice were used for scRAN-seq and scBCR-seq.

### scRNA-seq library preparation (10 × Genomics)

scRNA-seq libraries were generated with the 10 × Chromium platform 3’ v3 or 5’ library preparation kits (10 × Genomics) following the manufacturer’s instructions. Briefly, Single-cell Gel Beads-in-emulsion (GEMs) were formed on the Chromium Controller. The reverse transcription of barcoded RNA was performed. In this step, each cell was labeled with a specific barcode, and each transcript was labeled with a unique molecular identifier (UMI). Next, cDNAs were purified and amplified. Then the libraries were constructed by performing the following steps: fragmentation, ligation of sample index, adaptor ligation, SPRIselect cleanup, sample index PCR, and SPRIselect size selection. Purified libraries were sequenced with Illumina NovaSeq.

### scBCR-seq library preparation (10 × Genomics)

The samples were processed for single B-cell receptor (BCR) V(D)J clonotype with Chromium Single Cell 5’ Library & Gel Bead Kit v2 (PN-1000006). In short, Gel Beads-in-Emulsion (GEMs) were formed in channels of a chip in the 10 × Chromium instrument. GEM reverse transcription reactions were performed and the GEMs were cleaned up. Purified cDNAs were subjected to amplification before cleaning up with SPRIselect beads. BCR target enrichments were performed on full-length cDNA. Amplified cDNA underwent two rounds of target enrichment using nested primer pairs specific for mouse BCR constant regions. Single-cell BCR V(D)J libraries were prepared as recommended by the manufacturer’s user guide. The cDNA libraries were sequenced by Illumina NovaSeq.

### Read alignment and gene quantification

The fastq files were aligned to the mouse genome (GRCm38 version 3.0.0), which was downloaded from 10 × Chromium website (https://support.10xgenomics.com/single-cell-geneexpression/software/downloads/latest), using Cell Ranger (3.1.0) with the ‘count’ option and default parameters. After mapping, samples were grouped into individual AnnData objects by concatenating the raw_feature_bc_matrix_h5.h5 and adding the appropriate metadata information.

### Data pre-processing

Scrublet [90] (version 0.2.1) was used to determine the doublets in the datasets. Each cell was assigned a doublet score (scrublet_score), which was a score between 0 and 1. These predicted doublet cells as well as the scrublet_score were clustered and visualized using the UMAP method.

For quality control, we filtered the cells with higher than 13% of mitochondrial contents and cells of fewer than 500 UMIs or fewer than 200 features. Besides, we removed the cells predicted as doublets. Features expressed in fewer than 3 cells were removed in the analysis. We also removed the mitochondrial genes and ribosomal protein coding genes in the analysis. Cells expressing macrophage (*Cd68*), T cell (*Cd3d*, *Cd3e*), or red blood cell (*Hbb-bs*, *Hba-a1*) markers were excluded and left B cells (~ 82.8%) were used for the further analysis.

### Analysis and visualization of scRNA-seq data

SCANPY [91] was used for data analysis and visualization. The following steps were performed in an order: data normalization, log-transformation and highly variable genes selection using the ‘seurat’ flavor and principal component analysis. SCANPY as well as seaborn [92] was used for data visualization of heatmap, violin plot, etc.

### Identification of highly variable genes and cell clustering

We selected the highly variable genes using the highly_variable_genes function in SCANPY with parameters: min_mean = 0.015, max_mean = 10, min_disp = 0.05. 3, 904 highly variable genes were used for the data visualization. Harmony [93] (version 0.0.5) was used to integrate data from different samples. ‘Sample’ information in the metadata was corrected in two rounds of harmony iterations. Uniform Manifold Approximation and Projection (UMAP) [94] visualization and Louvain clustering [95] were performed based on the armony corrected latent space. The Louvain function in SCANPY was used with default parameters, while the resolution of the clustering change from 0.5 to 2. SCCAF [96] was used to determine the discriminative ability of the clusters.

### Differential gene expression analysis of scRNA-seq data

For differential gene expression analysis, the hurdle model in MAST [97] (version 1.12.0) was used to model the differential expression of the cells as well as the total number of counts. The genes with a false detection rate (FDR) lower than 0.01 were used for volcano plot. The genes with log fold change > 0.1 were considered as up-regulated genes, while others were the down-regulated genes.

### Re-visualization

For B1 cell development trajectory, we used Palantir [53] to derive the UMAP. In this process, Palantir first calculated the principal component space based on the highly variable genes and measured the diffusion map [98] using the principal component space. UMAP was then calculated on the resulted diffusion map space.

### Pseudotime analysis

The DPT function [98] in SCANPY was used for pseudotime analysis, while the start time point was assigned as a cell in the precursor cluster. The DPT function infered the progression of cells through geodesic distance along the graph [98, 99]. The Diffusion Pseudotime was initially introduced by Haghverdiand [98]. The implementation within Scanpy [91] used a further developed version, which was able to deal with disconnected graph [99].

### Single-cell gene signature scoring

To understand the gene expression in certain pathways, we used the score_genes function in SCANPY. The score is the average expression of a set of genes subtracted with the average expression of a reference set of genes, while the reference set is randomly sampled. B1 and B2 cell precursors signatures were from the previous study [15]—Table S1, which listed the genes differentially expressed in Lin^−^CD93^+^CD19^+^CD45R^−/low^ B1 progenitors and Lin^−^CD93^+^CD19^−^CD45R^+^CD43^+^ B2 progenitors, the DEGs that upregulated in day 2 neonate B1 progenitors and B2 progenitors were used.

### The analysis of scBCR-seq data

The fastq files from the BCR results were aligned to the mouse vdj reference (version 4.0.0) which was downloaded from the 10 × Chromium website using Cell Ranger (3.1.0) with the ‘vdj’ option and default parameters. The basic scBCR-seq statistics was then analysed using pyvdj v0.1.2. (https://github.com/veghp/pyVDJ). For BCR V(D)J data, only cells with at least one productive *Igh* chain and at least one productive *Igk or Igl* chain were considered for analysis. Immunarch [100] was used for further BCR analyses, including clonotype tracking, diversity estimation, repertoire overlap, gene usage analysis and chain pair analysis. We followed the same analysis workflow as presented in the tutorials at https://immunarch.com/.

### D50 analysis

To understand the presence of clonotypic expansions, we calculated the diversity 50 (D50) [101], which calculated percentage of dominant unique clones that account for 50% of the total clones. A higher D50 value means a higher diversity of the CDR3 sequence.

### Heavy and light chain pairing

We considered contigs identified as either heavy (Ighv) or light (Igkv/Iglv) chain variable domains. Identified variable domains were considered complete if both of the chains were found in the same cell (with the same cell barcode). We estimated the numbers of Ighv chains and the Igkv/Iglv chains, while the top 20 chain types were used for Ighv or Igkv/Iglv analysis. The jointplot function in seaborn [92] was used to estimate the heavy and light chain pairs and to plot the heatmap for the distributions.

### Flow cytometry and cell sorting

Cells were suspended in a solution of 2% (volume/volume) FBS in DPBS. Anti-mouse CD16/32 antibody (101,320, Biolegend) was used to block Fc receptors. Then cells were stained on ice for 30 min before washing. Intracellular staining was performed according to the manufacturer protocol of the FOXP3 Fix/Perm Buffer set (421,401, 421,402, Biolegend). Fluorochrome-labeled monoclonal antibodies (clones denoted in parentheses) against B220 (RA3-6B2), CD19 (6D5), CD3ε (145-2C11), CD4(RM4-5), CD8α (53–6.7), NK-1.1 (PK136), Nkp46 (29A1.4), CD11b (M1/70), CD11c (HL3), Gr1(RB6-8C5), Ter119 (TER-119), CD20 (SA271G2), IL-7Rα (A7R34), CD93 (AA4.1), MHCII (M5/115.15.2), CD138 (281–2), Sca-1 (D7), C-kit (2B8), CD43 (S11), CD5 (53–7.3), CD23 (B3B4), Ki67 (16A8), CD25 (PC61), Ly6D (49-H4) and CD36 (HM36) were purchased from BD Biosciences, Biolegend or eBioscience. Cells were analyzed on the LSRFortessa X-20 cell analyser (BD) or sorted on the FACSArial Fusion cell sorter (BD). Data was analyzed with FlowJo software (BD).

### Adoptive transfer

The cell populations were stained and sorted by flow cytometry, and were subsequently injected intravenously or intraperitoneally into sub-lethally irradiated (1 × 450 rads).

*Rag2*^*−/−*^ mice. The drinking water was added antibiotics for 2 weeks. The peritoneal cells from recipients were collected for analysis after 2–4 weeks injection.

### Statistical analysis

The statistical analysis was conducted with Microsoft Excel. P values were calculated using a two-tailed Student’s t-test.

## Supplementary Information

**Additional file 1:** **Figure S1. a** The violin plots showing the number of genes, the number of counts and the percentage of mitochondrial genes in all examined samples. **b** The violin plot showing the percentage of mitochondrial genes in each sample. **c** The scatter plot showing that the logarithm of the number of genes and the logarithm of the number of counts are linearly correlated. **d** UMAP plots showing the distribution of the six samples, the number of counts, the number of genes, and the percentage of mitochondrial genes. **e** The UMAP plots showing the distribution of the re-clustered samples after quality control. **f** The UMAP plots showing the marker gene expression in macrophages (*Cd68*, *S100a8*), T cells (*Cd3d*, *Cd3e*), erythrocytes (*Hbb-bs*, *Hba-a*1) in all examined samples. The color key indicates the expression level. **g** The UMAP plots showing the expression of the known B cell marker genes. The color key indicates the expression level. **Figure S2. a** The bar plot showing the percentages of day 5 and 6 neonate samples in each of the C1-C9 clusters. **b** The expression patterns of the MHCII molecules related genes and CD138 encoding gene in UMAP plot. The color key indicates the expression level. **c** Projection of the B2 cell progenitor signature (B2 cell progenitor signature was from a previous study, see more details in Methods). The color key indicates the average log normalized expression of the B1 signature genes over the randomly sampled reference genes. **d** The dot plot demonstrates cluster-specific gene expression in C1, C2, and C4 cells. **e**, **f** The expression pattern of the indicated genes in UMAP plot. The color key indicates the expression level. **g** FACS plots showing C1-C4 cells were present in neonate peritoneal cavity. Neonates (day 5–6, pool of 12 pups from 1 litter, n = 3) were used for analysis. **h** FACS plots showing the gating strategy of neonate peritoneal Lin^−^CD19^−^C-kit^low^Sca-1^low^ cells. Neonates (day 6, pool of 12 pups from 2 litters, n = 3) were used. Lineage markers: CD3ε, CD4, CD8α, NK-1.1, Nkp46, CD11b, CD11c, Gr1, Ter119. Data are representative of three (g, h) independent experiments. **Figure S3. a** The C5-C9 cells are highlighted in the UMAP projection. **b**, **c** Distribution of the indicated gene expression in UMAP plots. The color key indicates the expression level. **d** Table shows the identification markers for B cell subsets (C1-C9) in neonate peritoneal cavity. **Figure S4. a** The proportion of B1-C5 cluster in B1 cells in each mouse sample. **b** Bar plot showing the pathway analysis of DEGs that up-regulated (log fold change > 0.1, FDR < 0.01, n = 1, 468 genes) in the B1-C5 cells comparing to B1-C3 cells. The top 10 pathways are presenting. The gene set analysis tool GeneAnalytics (LifeMap Sciences) was used for DEGs pathway analysis. **c** Distribution of the aging-related gene expression in UMAP plots. The color key indicates the expression level. **Figure S5. a** The table lists the usages of V_H_ and V_L_ genes of both B1 and B2 cells from differently aged mice. **b** Bar plot shows the percentages of B1 cell clones that recognize the phosphatidylcholine (both *Ighv12-3/Igkv4-91* and *Ighv11-2/Igkv14-126* pairings) in examined mature B1 cells from mice of different age. **c** The expression pattern of the proliferation-related genes in the re-projected mature B1 cell UMAP plot. The color key indicates the expression level. **d** Bar plot shows percentages and numbers of the B1 cell clones that recognize the phosphatidylcholine (PtC) in mature proliferating B1 subset (B1-C4) in mice of different age.

**Additional file 2: Table S1.** the signature genes list.

**Additional file 3: Table S2.** DEGs up-regulated in C1 cells (C1 vs. C2).

**Additional file 4: Table S3.** DEGs up-regulated in C2 cells (C2 vs. C1).

**Additional file 5: Table S4.** DEGs up-regulated in C3 cells (C3 vs. C4).

**Additional file 6: Table S5.** DEGs up-regulated in C4 cells (C4 vs. C3).

**Additional file 7: Table S6.** DEGs up-regulated in B1-C5 cells (B1-C5 vs. B1-C3).

**Additional file 8: Table S7.** DEGs down-regulated in B1-C5 cells (B1-C5 vs. B1-C3).

## Data Availability

The scRNA-seq and scBCR-seq data described in this study have been deposited in ArrayExpress (https://www.ebi.ac.uk/arrayexpress/) under accession number E-MTAB-10081. All other relevant data are available from the corresponding author on request.

## Abbreviations

BCR: B-cell receptor
CDR-H3: Complementarity determining region 3 of the heavy chain
scRNA-seq: Single-cell RNA-sequencing
scBCR-seq: Single-cell B-cell receptor sequencing
UMAP: Uniform manifold approximation and projection
PtC: Phosphatidylcholine
drHSC: Developmentally restricted hematopoietic stem cell
UMI: Unique molecular identifier
FDR: False detection rate
DEGs: Differentially expressed genes
