# Supplementary material for: Single-cell genomics identifies distinct B1 cell developmental pathways and reveals aging-related changes in the B-cell receptor repertoire
Source: Cell Biosci. 2022 May 7;12:57. doi: 10.1186/s13578-022-00795-6 (PMC9080186; doi:10.1186/s13578-022-00795-6)

Figure S1

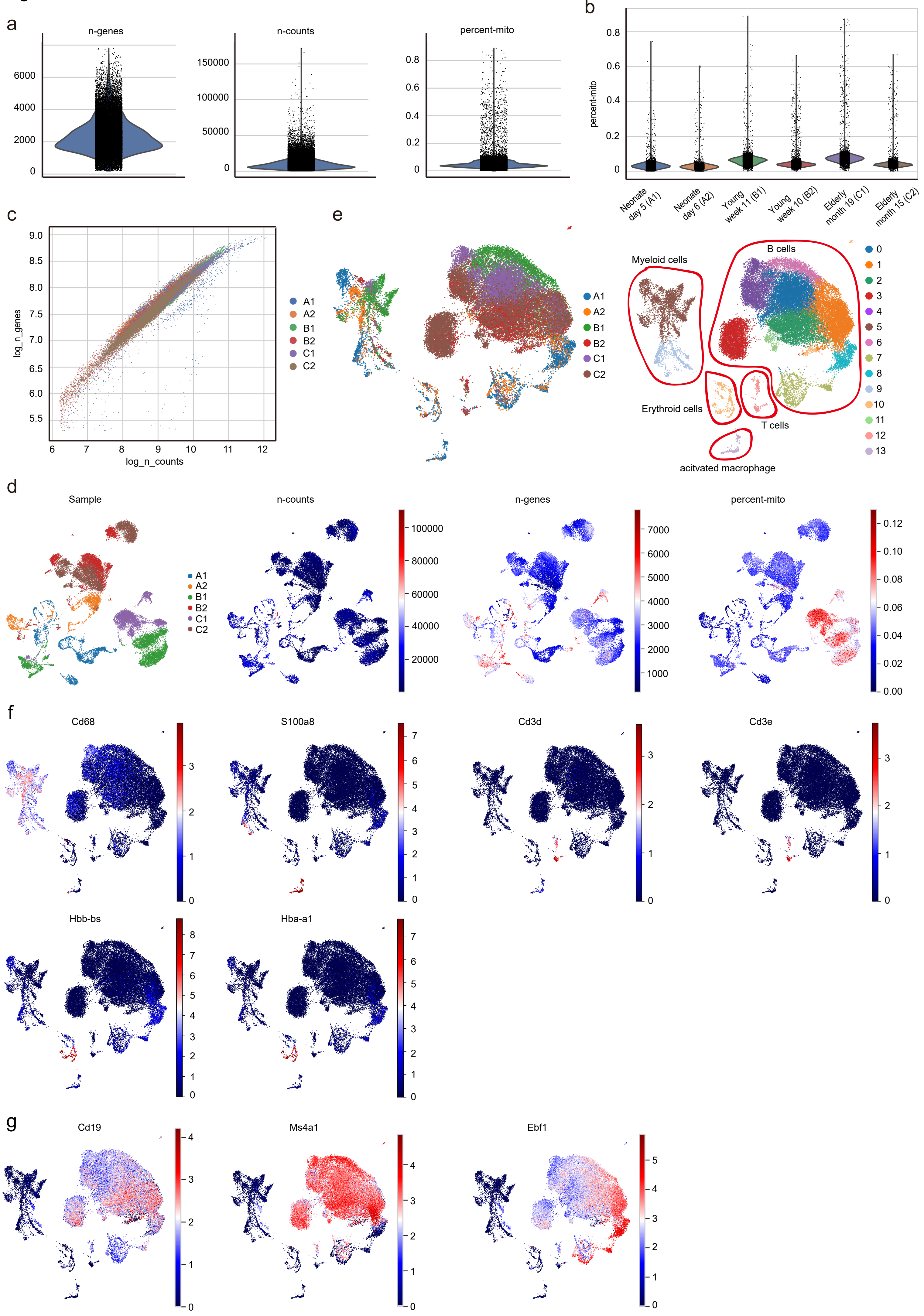
a
n-genes
n-counts
percent-mito
b
percent-mito
Neonate day 5 (A1)
Neonate day 6 (A2)
Young week 11 (B1)
Young week 10 (B2)
Elderly month 19 (C1)
Elderly month 15 (C2)
c
log_n_genes
log_n_counts
A1
A2
B1
B2
C1
C2
e
Myeloid cells
B cells
Erythroid cells
T cells
acitvated macrophage
d
Sample
n-counts
n-genes
percent-mito
f
Cd68
S100a8
Cd3d
Cd3e
Hbb-bs
Hba-a1
g
Cd19
Ms4a1
Ebf1

Figure S2

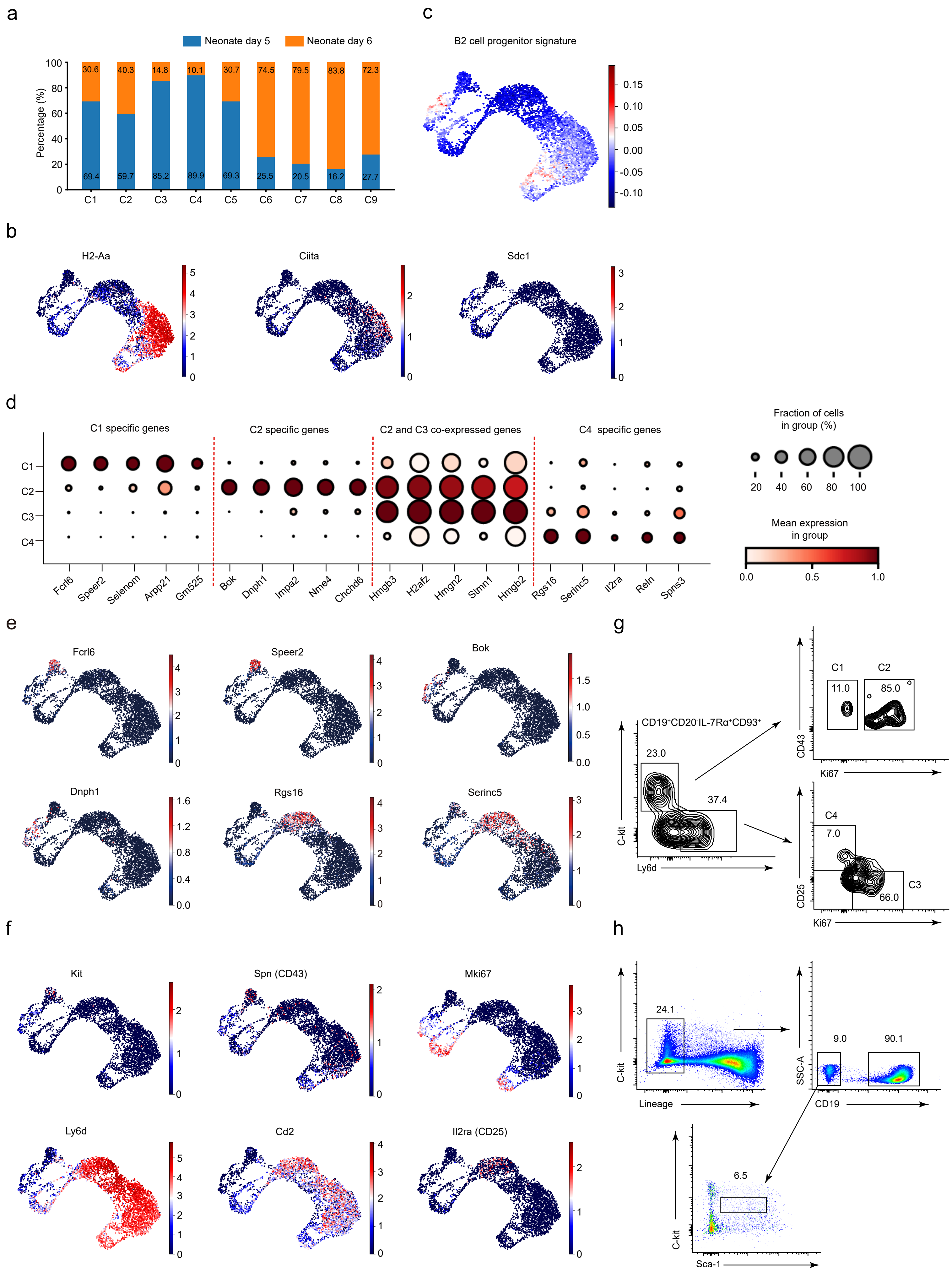
a
Neonate day 5
Neonate day 6
Percentage (%)
C1 C2 C3 C4 C5 C6 C7 C8 C9
c
B2 cell progenitor signature
b
H2-Aa
Ciita
Sdc1
d
C1 specific genes
C2 specific genes
C2 and C3 co-expressed genes
C4 specific genes
Fraction of cells in group (%)
Mean expression in group
Fcrl6 Speer2 Selenom Arpp21 Gm525 Bok Dnph1 Impa2 Nme4 Chchd6 Hmgb3 H2afz Hmgn2 Stmn1 Hmgb2 Rgs16 Serinc5 Il2ra Reln Spns3
e
Fcrl6
Speer2
Bok
Dnph1
Rgs16
Serinc5
f
Kit
Spn (CD43)
Mki67
Ly6d
Cd2
Il2ra (CD25)
g
CD19+CD20-IL-7Rα+CD93+
C-kit
Ly6d
CD43
Ki67
CD25
h
Lineage
SSC-A
CD19
Sca-1

Figure S3

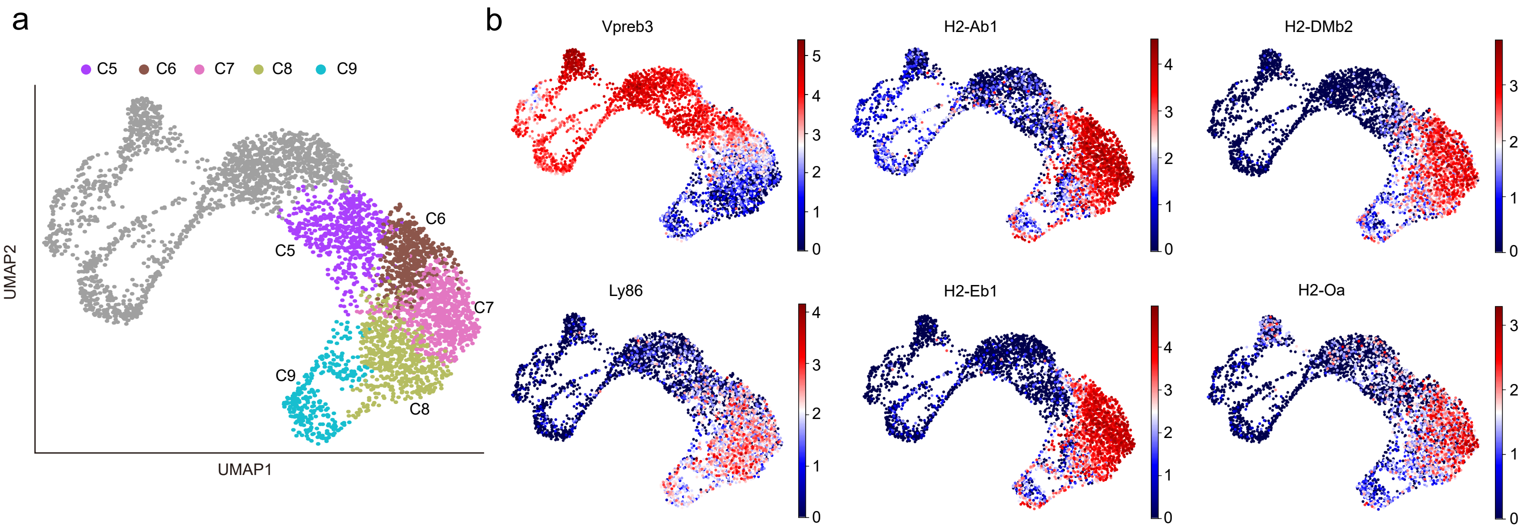

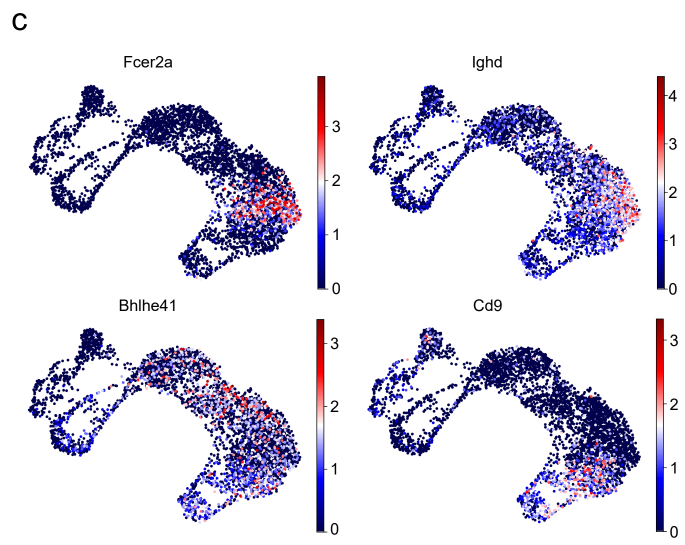

d

Cluster	Cell type	Identification markers
C1	Non-proliferating pro-B cells	$CD19^{+}CD20^{-}IL\text{-}7R\alpha^{+}CD93^{+}CD43^{+}C\text{-}kit^{+}Ki67^{-}$
C2	Proliferating pro-B cells	$CD19^{+}CD20^{-}IL\text{-}7R\alpha^{+}CD93^{+}CD43^{+}C\text{-}kit^{+}Ki67^{+}$
C3	Proliferating pre-B cells	$CD19^{+}CD20^{-}IL\text{-}7R\alpha^{+}CD93^{+}Ly6d^{+}CD25^{-}Ki67^{+}$
C4	Non-proliferating pre-B cells	$CD19^{+}CD20^{-}IL\text{-}7R\alpha^{+}CD93^{+}Ly6d^{+}CD25^{+}Ki67^{-}$
C5	Immature B cells	$CD19^{+}CD20^{+}CD93^{+}MHCII^{-}$
C6	Immature B cells	$CD19^{+}CD20^{+}CD93^{+}MHCII^{+}$
C7	B2 cells	$CD19^{+}CD20^{+}CD93^{-}CD23^{+}IgD^{hi}$
C8	B1 cells	$CD19^{+}CD20^{+}CD93^{-}CD23^{-}Bhlhe41^{+}Ki67^{-}$
C9	Proliferating B1 cells	$CD19^{+}CD20^{+}CD93^{-}CD23^{-}Bhlhe41^{+}Ki67^{+}$

Figure S4

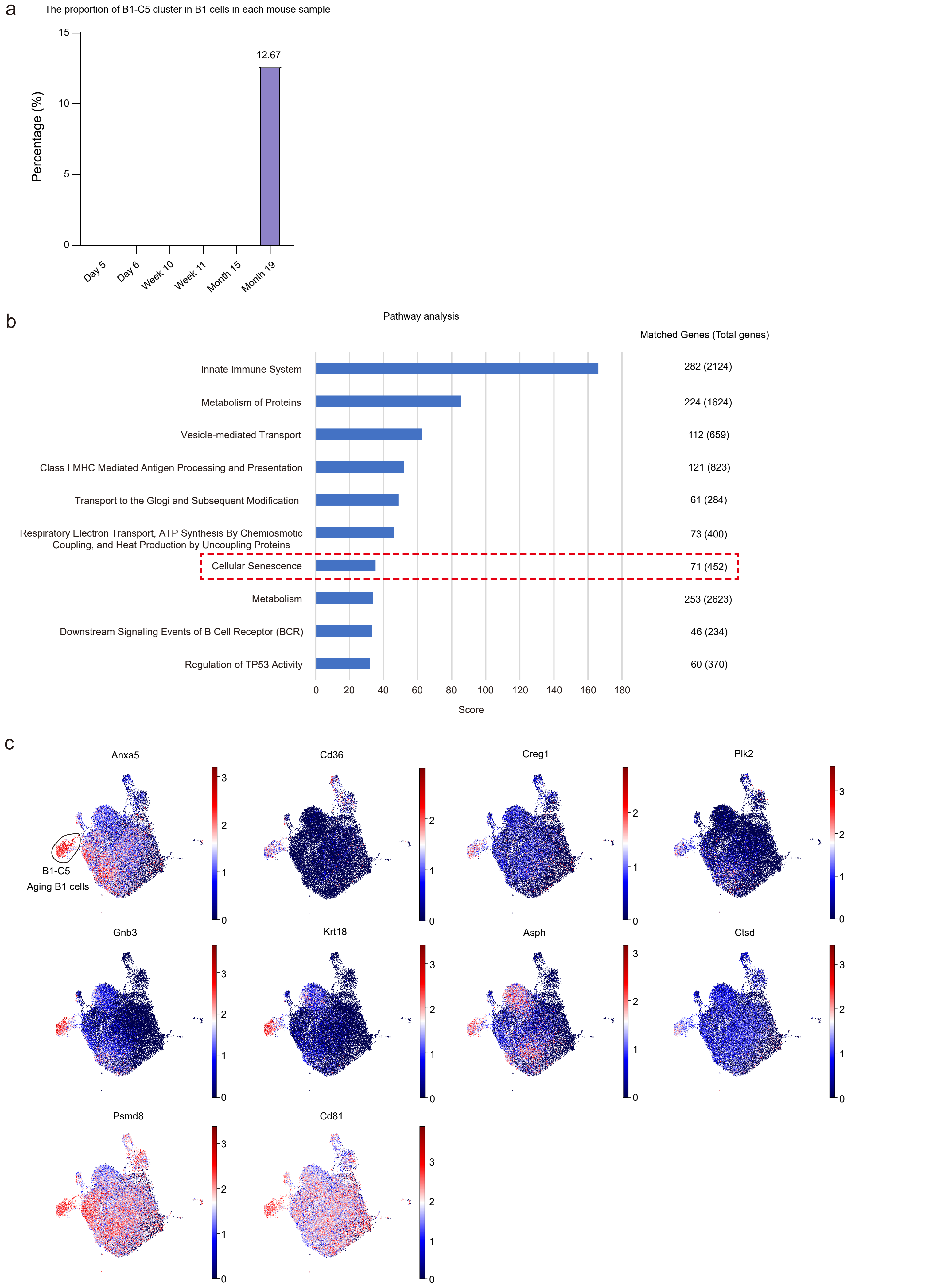

a
The proportion of B1-C5 cluster in B1 cells in each mouse sample
Percentage (%)
15
10
5
0
12.67
Day 5
Day 6
Week 10
Week 11
Month 15
Month 19
b
Pathway analysis
Matched Genes (Total genes)
Innate Immune System
282 (2124)
Metabolism of Proteins
224 (1624)
Vesicle-mediated Transport
112 (659)
Class I MHC Mediated Antigen Processing and Presentation
121 (823)
Transport to the Glogi and Subsequent Modification
61 (284)
Respiratory Electron Transport, ATP Synthesis By Chemiosmotic Coupling, and Heat Production by Uncoupling Proteins
73 (400)
Cellular Senescence
71 (452)
Metabolism
253 (2623)
Downstream Signaling Events of B Cell Receptor (BCR)
46 (234)
Regulation of TP53 Activity
60 (370)
0
20
40
60
80
100
120
140
160
180
Score
c
Anxa5
Cd36
Creg1
Plk2
B1-C5
Aging B1 cells
Gnb3
Krt18
Asph
Ctsd
Psmd8
Cd81

Figure S5

a

Cell type	Mouse age	Ighv	Igkv
B1 cells	day 6	813	854
	week 10	6,640	7,215
	month 15	7,193	8,234
B2 cells	day 6	686	719
	week 10	1,073	1,161
	month 15	925	1,064

c

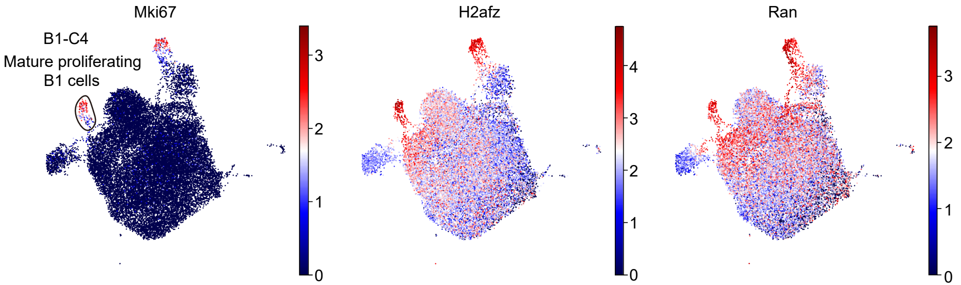

b

The proportion of anti-PtC cells (Ighv12-3/Igkv4-91 and Ighv11-2/Igkv14-126 pairings) in the total B1 cells

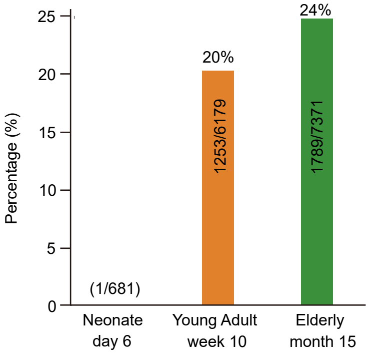

d

The proportion of anti-PtC cells in mature proliferating B1 subset

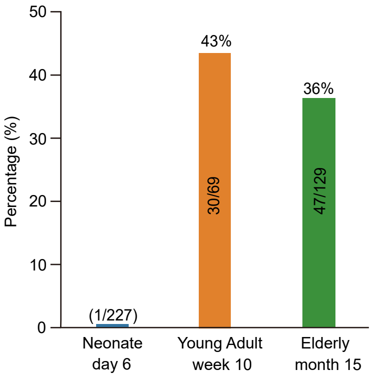

Supplement: Supplementary file 1 — Additional file 1: Figure S1. a The violin plots showing the number of genes, the number of counts and the percentage of mitochondrial genes in all examined samples. b The violin plot showing the percentage of mitochondrial genes in each sample. c The scatter plot showing that the logarithm of the number of genes and the logarithm of the number of counts are linearly correlated. d UMAP plots showing the distribution of the six samples, the number of counts, the number of genes, and the percentage of mitochondrial genes. e The UMAP plots showing the distribution of the re-clustered samples after quality control. f The UMAP plots showing the marker gene expression in macrophages (Cd68, S100a8), T cells (Cd3d, Cd3e), erythrocytes (Hbb-bs, Hba-a1) in all examined samples. The color key indicates the expression level. g The UMAP plots showing the expression of the known B cell marker genes. The color key indicates the expression level. Figure S2. a The bar plot showing the percentages of day 5 and 6 neonate samples in each of the C1-C9 clusters. b The expression patterns of the MHCII molecules related genes and CD138 encoding gene in UMAP plot. The color key indicates the expression level. c Projection of the B2 cell progenitor signature (B2 cell progenitor signature was from a previous study, see more details in Methods). The color key indicates the average log normalized expression of the B1 signature genes over the randomly sampled reference genes. d The dot plot demonstrates cluster-specific gene expression in C1, C2, and C4 cells. e, f The expression pattern of the indicated genes in UMAP plot. The color key indicates the expression level. g FACS plots showing C1-C4 cells were present in neonate peritoneal cavity. Neonates (day 5–6, pool of 12 pups from 1 litter, n = 3) were used for analysis. h FACS plots showing the gating strategy of neonate peritoneal Lin−CD19−C-kitlowSca-1low cells. Neonates (day 6, pool of 12 pups from 2 litters, n = 3) were used. Lineage mark [file 13578_2022_795_MOESM1_ESM.pdf]
